# Severity of restless leg syndrome in patients undergoing hemodialysis: assessing its burden on quality of life and knowledge

**DOI:** 10.3389/fneph.2026.1850856

**Published:** 2026-06-04

**Authors:** Esraa Samir Mohamed, Hanan Said Ali, Fatma Mostafa Mahrous

**Affiliations:** Faculty of Nursing, Ain Shams University, Cairo, Egypt

**Keywords:** Egypt, end-stage renal disease, hemodialysis, knowledge, patients, quality of life, restless leg syndrome

## Abstract

**Objectives:**

This study aimed to assess the severity of restless legs syndrome (RLS) among patients undergoing hemodialysis and to evaluate its burden on quality of life (QoL), as well as its relationship with knowledge regarding end-stage renal disease (ESRD), RLS, and hemodialysis (HD).

**Methods:**

In 2025, a descriptive correlation study was used. A purposive sample of (75) patients was recruited from the hemodialysis unit of Banha University Hospital in Qalyubia, Egypt. Self-administered questionnaires included: 1) socio-demographic characteristics; 2) a questionnaire assessing patient’s knowledge regarding ESRD, RLS, and HD; 3) the International Restless Legs Syndrome study group (IRLSSG) rating scale; and 4) A Quality of life (QoL) scale. Statistical analysis was analyzed using SPSS version 27.

**Results:**

A total of 75 patients were included, with a mean age of 47.6 ± 9.2 years; Of them, 65.3% were males, and 56% had been on dialysis for 5–10 years. Overall, 62.7% of the studied patients had an unsatisfactory level of knowledge regarding ESRD and RLS in the setting of hemodialysis. Regarding RLS severity, 10.7% had mild, 28.0% had moderate, 42.7% had severe, and 18.6% had very severe RLS. Overall, 48.0% of participants had poor total QoL. Overall RLS severity and QoL showed a statistically significant inverse association (p < 0.001). Overall RLS severity and QoL showed an inverse association with high statistical significance (p < 0.001). In addition, overall knowledge was inversely associated with overall RLS severity, though this did not reach statistical significance (p=0.056). However, QoL showed a statistically significant direct association with the overall level of knowledge (p < 0.029). Regarding QoL subdimensions, 52.0%, 54.7%, 49.3%, and 37.3% of patients reported poor QoL across the physical, psychological, social, and spiritual domains, respectively. Overall, 48.0% of participants had poor total QoL, 37.3% had average QoL, and 14.7% had good QoL.

**Conclusion:**

Among the investigated patients, QoL showed a negative association with overall RLS severity with high statistical significance. Moreover, overall knowledge was negatively and meaningfully associated with overall RLS severity. However, QoL was meaningfully and positively associated with the overall level of knowledge.

## 1 Introduction

Chronic kidney disease (CKD) is a significant contributor to illness and death worldwide in the 21st century (1). Its prevalence continues to increase, affecting approximately 800 million people globally in 2022 (2). A sustained glomerular filtration rate (GFR) of lower than fifteen mL/min/1.73 m² for a minimum of three months is indicative of end-stage renal disease, the final (stage five) phase of CKD (2). In the absence of renal replacement treatment (RRT), ESRD may result in severe and life-threatening complications. At this advanced stage, the kidneys are unable to support normal physiological demands. Therefore, RRT, including kidney transplantation, hemodialysis or peritoneal dialysis is required to maintain life (3).

The common treatment services for patients with end-stage renal disease (ESRD) include kidney transplantation and dialysis (4). Hemodialysis (HD) is a therapeutic procedure designed to eliminate excess fluids and toxins from the body when the kidney is incapable of performing this function, leading to the patient’s dependence on the treatment. HD is the most widely used and second-best treatment following kidney transplant that improves patients’ lifestyle and their quality of life for patients with renal failure (5).

Restless legs syndrome (RLS) is a common neurological illness, that may occur as a main issue or a side effect of different diseases (6). This syndrome is characterized by the uncomfortable or unusual feelings in the arms or legs accompanied by a desire to stretch the affected limbs. These manifestations typically arise during periods of rest, particularly at night, and movement may momentarily alleviate the symptoms (7). RLS negatively affects one’s quality of life (QoL) and is linked to mood disorders like depression and anxiety. QoL reflects the overall life satisfaction, feeling of wellbeing and the capacity to carry out daily activities (8).

The concept of quality of life (QoL) has gained recognition within the field of nephrology as a noteworthy patient-reported outcome measure (PROM) (9). Hemodialysis is time-consuming as patients must visit the blood purification center thrice a week for about four hours each time. Besides, HD patients must pay close attention to their diet and fluid intake, physical activity, and medication to maintain good treatment outcomes during hemodialysis (10). These place a heavy burden on patients and have a profound impact on their mental health and quality of life (11).

By 2030, the prevalence of CKD is projected to range from 8% to 16% of the global population, representing over 800 million individuals (12). According to the United States Renal Data System 2023 annual report, over 808,000 Americans are living with ESRD, with 68% undergoing hemodialysis and 32% having received a kidney transplant. International databases indicate that approximately 30% of hemodialysis patients are affected by RLS (12, 13).

In Egypt, the estimated prevalence of CKD was 7.1 million, or 106 cases per 1000 people. Furthermore, it is anticipated that by 2030, the number of CKD patients will double. RLS prevalence in Egypt is 22.32%, twice the rate of idiopathic RLS 12%, with kidney disorders at 26% and 10%, respectively (14, 15).

Improving QoL for HD patients with RLS may lead to reduce hospitalization rates and enhancing survival outcomes (16). Enhancing QoL may help alleviate treatment burdens and improve patient health outcomes. Furthermore, improved QoL can potentially lower long-term healthcare costs by decreasing mortality rates. In addition, better QoL is associated with improved physical and psychological functioning, which may positively influence patient productivity (17).

In summary, addressing RLS in HD patients is essential to enhancing their QoL and controlling related symptoms. RLS should be taken into account by healthcare professionals as part of ESRD patients’ overall care to improve their wellbeing both during and after dialysis Therefore, the primary objective of this study was to assess the relation between severity of RLS and QoL for patients with ESRD undergoing HD program.

## 2 Materials and methods

### 2.1 Study design and period

A descriptive correlational study design was conducted from May 2025 to August 2025 to assess the severity of restless legs syndrome (RLS) among patients undergoing hemodialysis and to evaluate its burden on quality of life (QoL), as well as its relationship with knowledge regarding end-stage renal disease (ESRD), RLS, and hemodialysis (HD).

### 2.2 Study setting

The study was conducted in the hemodialysis unit of Banha University Hospital in Qalyubia, Egypt, which is affiliated with the Ministry of Health and Population in Egypt. The HD unit on the second floor and comprises a physicians’ room, two rooms for nursing staff, and treatment rooms for HD patients which including room (A) for patients with negative hepatitis C virus (HCV) status and room (B) for patients with positive HCV status. Room A contains 21 hemodialysis machines, whereas room B contains 16. Approximately ten chairs are available in the waiting area outside the unit.

### 2.3 Study participants and sampling technique

A purposive sample of (75) patients diagnosed with restless leg syndrome (RLS) and end-stage renal disease (ESRD) and receiving hemodialysis (HD) was recruited for this descriptive correlational study. The sample size was determined using Power Analysis and Sample Size (PASS) software (Version 15.0.10). The confidence level was set at 90%, with a margin of error of ±0.10. Assuming a moderate correlation between the severity of RLS and quality of life (QoL) among patients with ESRD undergoing HD (r = 0.70), a minimum sample size of 75 participants was considered sufficient to achieve the study objectives.

### 2.4 Eligibility criteria

Participants were recruited using purposive sampling from HD patients with RLS and ESRD, of either gender, aged >18 years, who met the RLS diagnostic criteria assessed using the International Restless Legs Syndrome Study Group (IRLSSG) scale, consisting 4 items (18)., All participants were receiving HD via an arteriovenous shunt 2–3 times per week and were oriented and able to communicate with others. By contrast, patients receiving HD for acute renal failure and those with significant neurological disorders were excluded.

### 2.5 Data collection

In the current study, researchers distributed data collection tools to patients at HD units after the dean of Banha University Hospital’s hemodialysis unit gave authorization for data collection. Three instruments were employed for gathering data. The first is an interview-based questionnaire that was modified from recent relevant literature (18, 19) to assess the sociodemographic characteristics of the patients studied and the job characteristics and past medical history and current dialysis session data. The second is gauge how severe a patient’s RLS symptoms were. The third is to assess to evaluate the four aspects of QoL for those with ESRD on HD.

### 2.6 Part I: an interview-based questionnaire

This questionnaire was designed by the researchers based on a review of the related literature (18, 19) and was written in simple Arabic to gather data on the sociodemographic characteristics of the patients studied and the job characteristics of the study subjects, such as marriage status, age, gender, degree of education and employment situation and monthly income, and past medical history and current dialysis session data such as Dialysis sessions’ duration and frequency each week.

### 2.7 Part II: patient’s knowledge regarding ESRD, RLS, and HD questionnaire

In the current study, patient’s knowledge regarding ESRD, RLS, hemodialysis, exercise, diet and follow up survey was used. This tool was adapted from relevant related literature from (20–22) to assess patient’s knowledge regarding ESRD, RLS, HD, exercise, diet and follow up. It was written in Arabic to collect data.

#### 2.7.1 Scoring system

The patient’s knowledge regarding ESRD, RLS, and HD questionnaire consists of 32 items: multiple choice questions (MCQ). These items cover topics such as the about kidney function, definition of chronic kidney disease (CKD), glomerular filtration rate in ESRD, causes of ESRD, signs and symptoms of ESRD, diagnostic tests, and complications, etc. A key response to the model was categorized on the basis of the correctness of the responses. Each correct answer received a “one points” and incorrect answers received “zero point”. These points were then summed and converted into percentage scores. This total score was 32, and the knowledge levels were classified into two categories: unsatisfactory level was indicated by a total knowledge score < 75%, and A satisfactory level was indicated by a total knowledge score ≥ 75% (23).

### 2.8 Part III: international restless leg syndrome study group rating scale

This instrument was adopted from Walters et al. (24) to assess the severity of a patient’s RLS symptoms. It consists of 10 items, which included: 1) How the patient rates RLS discomfort in the legs or arms; 2) the need to move because of RLS symptoms; 3) the degree of relief from RLS discomfort after moving; 4) the severity of sleep disturbance due to RLS; 5) the severity of tiredness or sleepiness attributable to RLS; 6) the severity of RLS overall; 7) how often RLS symptoms occur; 8) the severity of RLS symptoms on an average day; 9) the extent to which RLS symptoms affect mood and the patient’s ability to carry out satisfactory family, home, social, or work life/overall daily activities; and (10) the severity of mood disturbance associated with RLS (e.g., angry, depressed, sad, anxious, or irritable).

#### 2.8.1 Scoring system:

The patient perception of the severity of RLS symptoms consists of 10 items. Participants rated each item using a four-point Likert scale from 0 = “Never” to 4 = “extremely severe”. Responses indicating “extremely severe” were classified as severe RLS, whereas responses indicating “Never” were classified as RLS absent. The total score reflects symptom severity across all domains and ranges from 0 - 40. This total score is translated to the corresponding RLs severity as follows:

0 indicated no RLS; 1–10 indicated mild RLS; 11–20 indicated moderate RLS; 21–30 indicated severe RLS; and 31–40 indicated extremely severe RLS.

### 2.9 Part IV: kidney disease quality of life short form

The Kidney Disease Quality of Life Short Form (KDQoL-SF™) is a self-administered questionnaire originally developed by Wight et al. (25). In the present study, the KDQoL-SF™ version 1.3 was used (available at: https://www.rand.org/health-care/surveys_tools/kdqol.html), derived from the Medical Outcomes Study Questionnaire Short Form-36 (SF-36). The instrument was translated into Arabic and then back-translated to ensure accuracy. The questionnaire was adopted to evaluate the four aspects of QoL (physical, psychological, social and spiritual) in patients with ESRD receiving HD.

#### 2.9.1 Scoring system

In the current study, the KDQOL-SF consists of 36-items divided into four dimensions physical (16 items), psychological (10 items), social (6 items), and spiritual (4 items). Participants rated each item using a five-point Likert scale from 0 = “never” to 5 = “always”. The option “always” is considered a positive response, whereas “never” was considered a negative response. Item scores were then transformed to a 0–100 scale, where 0 represents the lowest quality of life and 100 represents the highest quality of life. The total scores of positive responses related to all domains are 100 scores. This total QoL scores was classified into three categories: < 50% indicating worst QoL, 50% - <75% indicating average QoL and ≥75% indicating best QoL (25).

### 2.10 Content validity and reliability

The validity of the face and content of the study instruments, including the patients’ knowledge questionnaire regarding ESRD, RLS, and HD questionnaire, the international RLS study group rating scale, and the Kidney Disease Quality of Life Short Form (KDQoL-SF) were established by a panel of six experts in medical–surgical nursing: three professors from the department of medical–surgical nursing, Ain Shams University, and three professors from the department of medical–surgical nursing at Benha University. Experts revised the tools for clarity, relevance, accuracy, and comprehensibility and minor modifications were made to produce the final forms. Because the original instruments were developed in English, cultural and linguistic adaptation was performed to ensure appropriateness for the Egyptian healthcare context. In addition, the reliability of the developed tools was tested using Cronbach’s alpha coefficients, demonstrating high internal consistency: the patients’ knowledge questionnaire (32 items) showed a Cronbach’s alpha of 0.870, the International RLS Study Group rating scale (10 items) showed 0.880, and the KDQoL-SF (36 items) showed 0.893.

### 2.11 Pilot study

A pilot study was conducted in May 2025 on 10% of the target sample size (8 patients) undergoing hemodialysis, to assess the extent to which the instruments are clarity, applicability, relevance, feasibility, and time-efficiency to estimate the time to fill it, which ranged between approximately 25 and 35 minutes. Based on the pilot results, no modifications were made and patients were added to the findings of the pilot study findings.

### 2.12 Ethics statement

A formal approval was obtained from the Research Ethic Committee of the Faculty of Nursing, Ain Shams University, Registered number 24.05.294, to carry out the study. Additionally, written informed consent was obtained from the nursing director of Benha Hospital and from all participating patients. Participants were informed that participation is voluntary and that they have the right to discontinue or withdraw from the study at any time without providing any reason.

### 2.13 Data analysis

The Shapiro-Wilk test was used to assess the normality of the primary continuous variables (RLS severity score, knowledge score, and QoL score). Given that RLS severity scores demonstrated a significant departure from normal distribution (Shapiro-Wilk p<0.05), Spearman’s rank-order correlation coefficient (r) was used to evaluate the relationships between variables (**Table 1**). The independent sample t-test was used to compare mean scores of continuous variables between two groups (e.g., knowledge scores by gender). The chi-square (χ²) test of independence was used to examine associations between categorical variables (e.g., knowledge level by education category). For all chi-square tests, the χ² value, degrees of freedom (df), and exact p-value are reported. The threshold for statistical significance was set at p<0.05, with p<0.01 indicating a highly significant result.

**Table 1:** Correlation between total level of knowledge, severity level of RLS and QoL among the studied patients.

Total level	QoL		Severity of RLS	
	r	p-value	r	p-value
Severity of RLS	-0.415	<0.001**		
Knowledge	0.528	0.029*	-0.296	0.056

r, Spearman Correlation Coefficient.

*Correlation is statistically significant at the p < 0.05*.

**Correlation is highly statistically significant at the p < 0.001**.

RLS, Restless Leg Syndrome; QoL, Quality of Life.

## 3 Results

### 3.1 Socio-demographic characteristics

**Table 2** reveals the socio-demographic characteristics of HD patients. A large percentage 62.7% of the patients studied were in the age group 50–70 years, with a mean age of 47.6 ± 9.2 years; 65.3% of them were males, 77.3% of them were married, and had intermediate education 52.0%, Regarding employment status, 72.0% of patients were not working, and 77.3% reported that their monthly income was insufficient to cover treatment costs. In terms of past medical history, 49.3% of the studied patients had been diagnosed with renal failure for 5–10 years, and 56.0% of them had been on dialysis for 5–10 years. Additionally, 90.7% underwent three dialysis sessions per week, and 82.7% had dialysis sessions lasting 4 hours (**Table 2**).

**Table 2:** Frequency and percentage distribution of HD patients according to their socio-demographic characteristics, past medical history and current dialysis session data (n=75).

Variables		n (%)
Age	18–30 years	5 (6.6)
	30–50 years	20 (26.7)
	50–70 years	47 (62.7)
	≥ 70 years	3 (4.0)
	Mean ± SD	47.6 ± 9.22
Gender	Male	49 (65.3)
	Female	26 (34.7)
Marital status	Married	58 (77.3)
	Unmarried	17 (22.7)
Education level*	Can’t read/write	7 (9.3)
	Basic	18 (24.0)
	Intermediate	39 (52.0)
	Higher	11 (14.7)
Occupation	Work	21 (28.0)
	Not work	54 (72.0)
Monthly income is enough to cover costs of treatment	Enough	17 (22.7)
	Not Enough	58 (77.3)
Duration of disease	1–5 years	19 (25.3)
	5–10 years	37 (49.3)
	10–15 years	15 (20.0)
	15 years and above	4 (5.3)
Duration of dialysis	1–5 years	17 (22.7)
	5–10 years	42 (56.0)
	10–15 years	13 (17.3)
	≥15 years	3 (4.0)
Number of dialysis sessions per week	1 per week	0 (0.0)
	2 per week	7 (9.3)
	3 per week	68 (90.7)
Duration of dialysis session	2 - <3 hrs.	0 (0.0)
	3 - <4 hrs.	5 (6.6)
	4 hrs.	62 (82.7)
	> 4 hrs.	8 (10.7)

Data are expressed as percentages for categorical variables. SD, Stander Deviation; n, number; (%), percentage; HD, Hemodialysis; hrs., hours.

*Education level: Basic, primary education; Intermediate, secondary education; Higher, college/university education.

### 3.2 Patient’s knowledge regarding ESRD, RLS, and HD

**Table 3** displays the distribution of HD patients by knowledge domain. 69.3% of the studied patients had unsatisfactory level of knowledge about ESRD and 65.3% of the studied patients had unsatisfactory level of knowledge about nutrition. Additionally, 61.3% of the studied patients had unsatisfactory level of knowledge about severity RLS (**Table 3**).

**Table 3:** Frequency and percentage distribution of HD patients according to their knowledge domains.

Knowledge domains	Satisfactory	Unsatisfactory
	n (%)	n (%)
Knowledge about ESRD	23 (30.7)	52 (69.3)
Nutrition	26 (34.7)	49 (65.3)
Knowledge about severity of RLS	29 (38.7)	46 (61.3)
Knowledge about dialysis	31 (41.3)	44 (58.7)
Sports and exercises	33 (44.0)	42 (56.0)
Follow-up and periodic check-ups	34 (45.3)	41 (54.7)

Data are expressed as percentages for categorical variables. SD, Standard Deviation; HD, Hemodialysis; RLS, Restless Legs Syndrome; ESRD, End-Stage Renal Disease.

**Figure 1:**
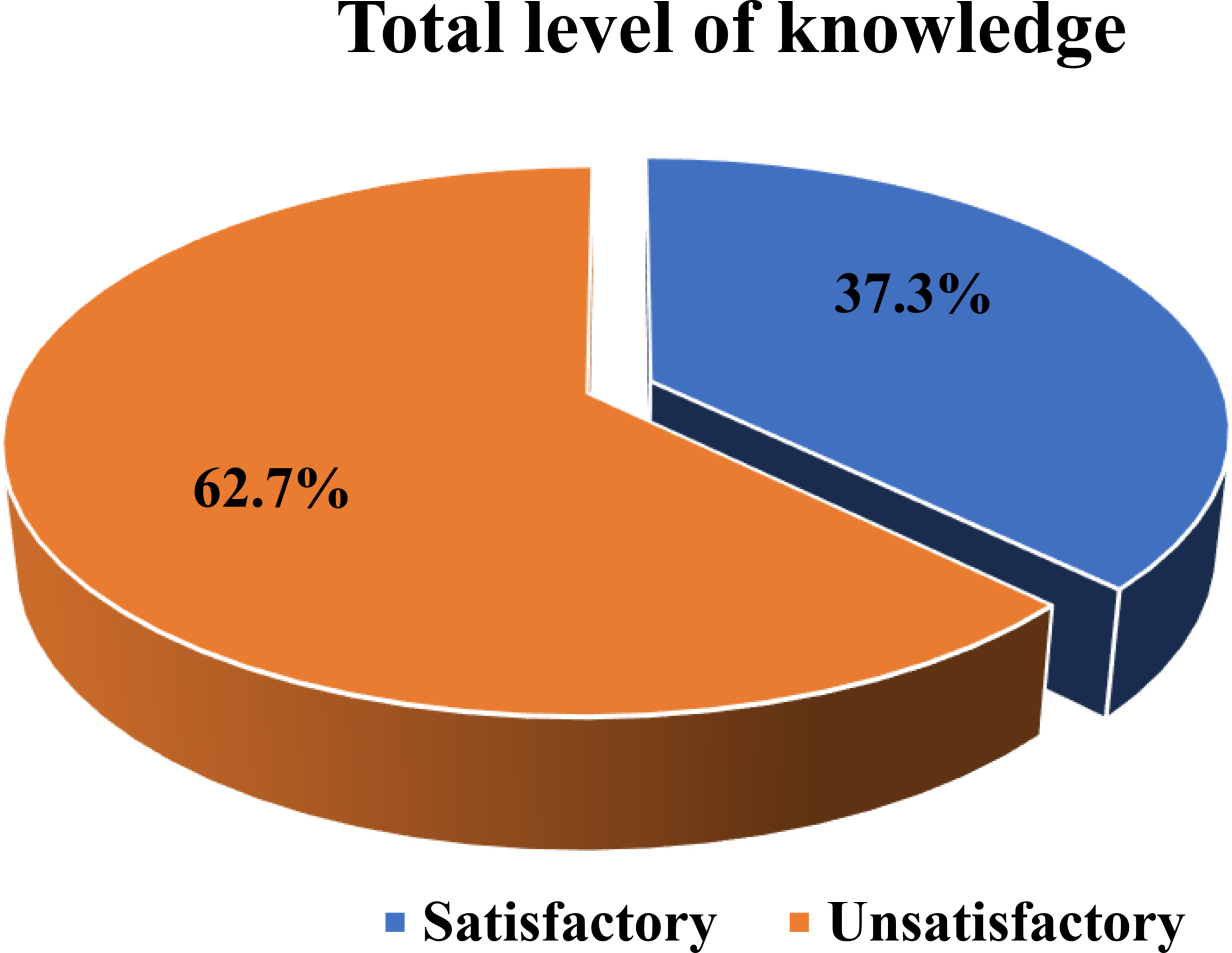
Distribution of hemodialysis patients by overall knowledge of RLS and ESRD (n = 75).

**Figure 1** presents the distribution of HD patients by overall knowledge of RLS and ESRD. Overall, 37.3% of participants had a satisfactory level, whereas 62.7% had an unsatisfactory level.

**Table 4** displays the distribution of HD patients according to their RLS severity. Among the participants, 52.0% reported severe sleep disturbance, and 50.7% reported severe impact of RLS symptoms on mood disturbance. Regarding the overall severity of RLS, 57.3% of participants reported a moderate level, as presented in (**Table 4**).

**Table 4:** Frequency and percentage distribution of HD patients according to their severity of (RLS).

Items	None	Mild	Moderate	Severe	Very severe
	n (%)	n (%)	n (%)	n (%)	n (%)
RLS discomfort in legs or arms	0 (0.0)	12 (16.0)	20 (26.7)	34 (45.3)	9 (12.0)
The need to move around	12 (16.0)	13 (17.3)	31 (41.3)	14 (18.7)	5 (6.7)
Sleep disturbance	4 (5.3)	9 (12.0)	12 (16.0)	39 (52.0)	11 (14.7)
Tiredness or sleepiness	5 (6.7)	9 (12.0)	16 (21.3)	32 (42.7)	13 (17.3)
Overall, severity of RLS	0 (0.0)	5 (6.7)	43 (57.3)	15 (20.0)	12 (16.0)
Number of times RLS symptoms appear per week.	0 (0.0)	13 (17.3)	15 (20.0)	31 (41.3)	16 (21.3)
Number of hours RLS symptoms occurs on an average day.	0 (0.0)	15 (20.0)	30 (40.0)	18 (24.0)	12 (16.0)
Impact of RLS symptoms on ability to carry out a satisfactory family, home, social or work life/overall daily affairs	4 (5.3)	8 (10.7)	18 (24.0)	29 (38.7)	16 (21.3)
Impact of RLS symptoms on mood disturbance (angry, depressed, sad, anxious, or irritable	3 (4.0)	11 (14.7)	13 (17.3)	38 (50.7)	10 (13.3)

Data are expressed as percentages for categorical variables. HD, Hemodialysis; RLS, restless leg syndrome.

RLS severity was classified according to the IRLSSG rating scale total score (range: 0–40), where 0, none, 1–10, mild, 11–20, moderate, 21–30, severe, and 31–40, very severe. Individual item scores (each 0–4) are presented separately for descriptive purposes and do not sum to the total-score severity classification.

### 3.3 Severity restless leg syndrome

**Figure 2:**
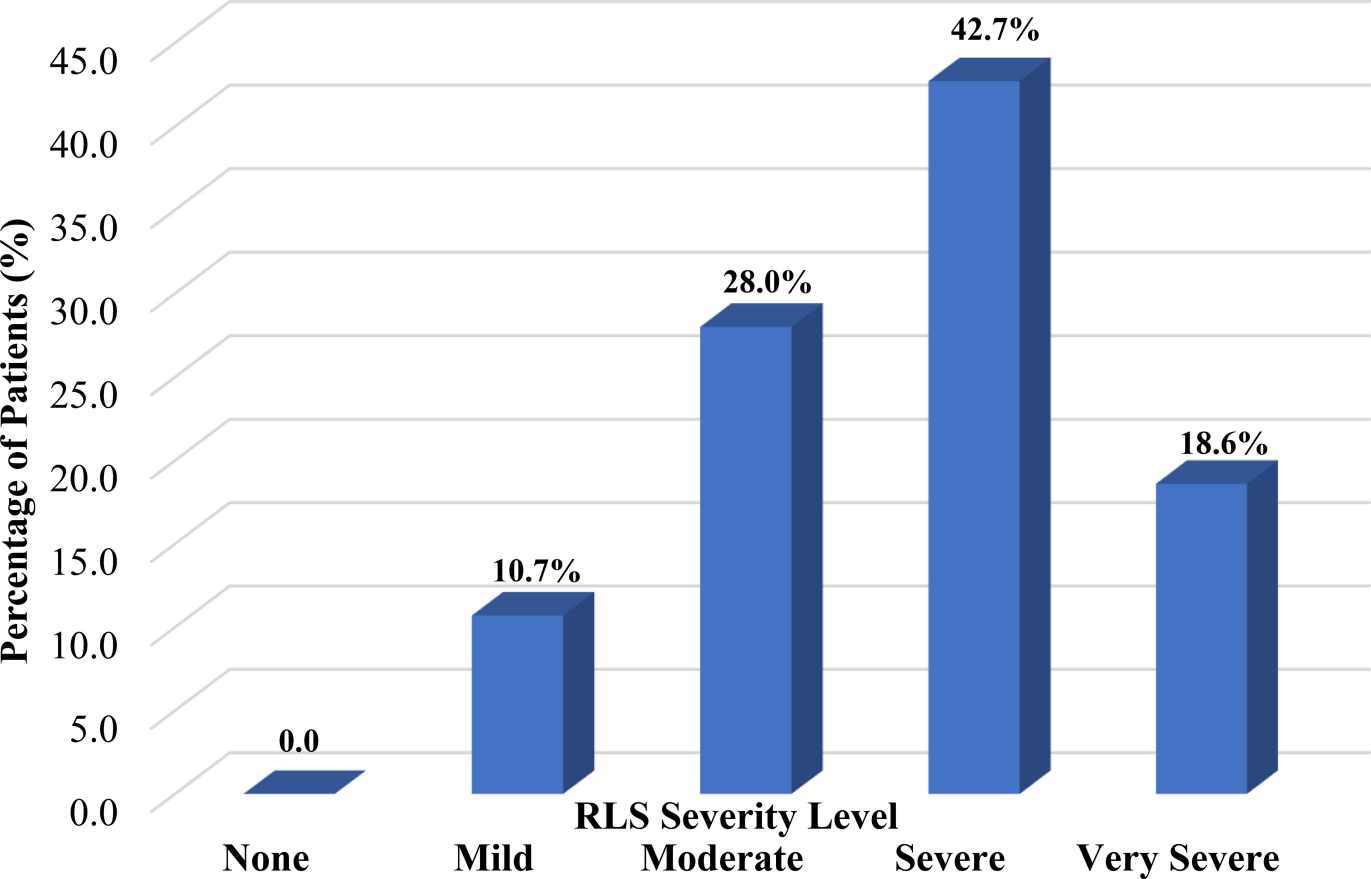
Percentage distribution of hemodialysis patients by total restless legs syndrome (RLS) severity level (n = 75).

**Figure 2** shows the overall percentage distribution of hemodialysis patients by total RLS severity level (n = 75). Overall, 42.7% of the participants had severe RLS and 28.0% had moderate RLS. In addition, 18.6% had very severe RLS, whereas only 10.7% had mild RLS; none had none/missing RLS severity.

### 3.4 Kidney disease quality of life

**Table 5** displays the distribution of Quality of Life (QoL) subdimensions by frequency and percentage. Regarding the physical, psychological, and social dimensions, 52.0%, 54.7%, and 49.3% of patients reported poor QoL, respectively. In the spiritual dimension, 37.3% of patients reported poor QoL, 44.0% reported average QoL, and 18.7% reported good QoL. With respect to overall QoL, the majority of patients rated their status as poor (48.0%) or average (37.3%), while only a small minority (14.7%) reported good overall QoL (**Table 5**).

**Table 5:** Patients’ distribution by frequency and percentage based on their QoL subdimensions.

Subdimensions of QoL	Poor	Average	Good
	n (%)	n (%)	n (%)
Physical dimension	39 (52.0)	30 (40.0)	6 (8.0)
Psychological dimension	41 (54.7)	25 (33.3)	9 (12.0)
Social dimension	37 (49.3)	29 (38.7)	9 (12.0)
Spiritual dimension	28 (37.3)	33 (44.0)	14 (18.7)
Total level of Quality of life	36 (48.0)	28 (37.3)	11 (14.7)

Data are expressed as percentages for categorical variables. QoL, Quality of Life.

**Table 1** presents the correlations between the total level of knowledge, the severity of RLS, and QoL among HD patients. Overall RLS severity and QoL showed an inverse association with high statistical significance (p<0.001**). In addition, overall knowledge was inversely associated with overall RLS severity, though this did not reach statistical significance (p=0.056). However, QoL exhibited a statistically significant direct association with the overall level of knowledge (p<0.029*) (**Table 1**).

## 4 Discussion

In our study, the researchers discussed the relationship between restless legs syndrome (RLS) severity and quality of life (QoL) among patients with end-stage renal disease (ESRD) undergoing hemodialysis, by assessing the severity of RLS, evaluating QoL, and examining the association between these variables.

The final and irreversible stage of CKD is known as ESRD, which necessitates renal replacement therapy (RRT) like hemodialysis to maintain life. RLS manifests as a strong, uncontrollable desire to move the legs, frequently while sitting, sleeping, or resting, which interferes with sleep and lowers QoL. Patients with CKD may develop RLS due to uremia resulting from insufficient hemodialysis. For RLS patients, particularly those with ESRD, early diagnosis is crucial to preventing muscle atrophy and enhancing their QoL (26, 27).

The study’s sociodemographic data showed that 44 patients (58.7%), aged 50–70 years, had a mean age of 47.6 ± 9.22 years. This represents the peak period for ESRD diagnosis and hemodialysis initiation because of cumulative exposure to risk factors like diabetes and hypertension. Males comprised 49 patients (65.3%), which may be explained by the greater prevalence of these risk factors among men. Given that ESRD and RLS are more common in middle-aged to older adults, 56 patients (74.7%) were married. Regarding education, 39 patients (52.0%) had an intermediate degree of education, reflecting their low socioeconomic status. Additionally, 54 patients (72.0%) were unemployed due to the demands of regular hemodialysis sessions, and consequently, 58 patients (77.3%) reported insufficient monthly income to cover treatment costs. These findings were consistent with those reported by Ruggajo et al. (28), who found that, with an average age of 49.56 ± 0.69 years, nearly three-fifths of participants were aged 50–60 years. Similarly, Yehia et al. (29) reported that males constituted approximately three-quarters of participants. Additionally, Alosaimi et al. (2020) (30) indicated that nearly four-fifths were married and over one-third had a middle level of education. Moreover, research by Ahmed et la (31). reported that, half of the study population were unemployed, while El-Kass et al. (21) and Al-Khattabi (32) showed that over two-thirds of participants had low income.

Considering duration of disease and dialysis, 37 patients (49.3%) had been diagnosed with renal failure for five to less than ten years, while 42 patients (56.0%) had been on dialysis for the same period. This finding could be attributed to the fact that the studied patients were aged between 50–70 years. Individuals in this age group at higher risk of earlier renal failure diagnosis, partly because they often undergo more frequent health checkups and medical follow-up than younger individuals, which may lead to earlier initiation of dialysis. This finding is consistent with previous studies by Jahan et al. (33) in Bangladesh and Abu El-Kass et al. (34) in Palestine.

As regards current dialysis session data, 68 patients (90.7%) underwent three dialysis sessions per week, and 62 patients (82.7%) had sessions lasting approximately four hours. This observation may be related to the fact that a three-times-weekly regimen, with sessions of about four hours, is the internationally recommended schedule for achieving adequate solute clearance, maintaining fluid balance, and optimizing dialysis efficiency in most patients with ESRD. This finding is consistent with the results reported by El-Kass et al. (21) and Al-Khattabi (32).

Our study assessed the degree of ESRD knowledge among patients. The findings showed that 52 patients (69.3%) had inadequate information about ESRD. The researchers suggest that this may be related to the educational profile of the sample, as 39 patients (52.0%) had an intermediate level of education. Limited education can adversely affect health literacy, thereby making it more difficult for patients to understand medical information, identify symptoms, and adhere to treatment plans effectively. This result is consistent with that reported by (35), who found that over two-thirds of the participants had low CKD knowledge.

Regarding the degree of RLS knowledge among patients, 46 patients (61.3%) demonstrated inadequate RLS information. This finding may be explained by the fact that patient education in dialysis units typically emphasizes dialysis-related care and comorbidities rather than sleep disorders. In addition, RLS is often underdiagnosed and frequently misinterpreted as muscle cramps or neuropathy. This result aligns with the study by Yıldız et al. (36), which similarly reported that fewer than two-thirds of the participants had a low level of RLS awareness.

Regarding the degree of dialysis knowledge among patients, the study findings showed that 44 patients (58.7%) had inadequate dialysis information. From the investigator’s perspective, this could be connected to low health literacy, limited access to individualized education, the complexity of medical information and inconsistent follow-up care, all of which can hinder patients’ understanding of dialysis treatment. This outcome is consistent with the findings of Kameel Zatton et al. (23), who reported that 54.0% of the participants had inadequate dialysis information.

Concerning the degree of exercise-related knowledge among patients, the study findings indicated that 42 patients (56.0%) had inadequate information. This could be connected to limited health education, misconceptions about the safety of physical activity during illness, and insufficient of guidance from healthcare providers. Collectively, these factors can reduce patients’ awareness of both the potential benefits and the safety of exercise, especially in chronic conditions. This outcome is consistent the findings of Verma et al. (37), who reported that 63.0% of the patients had inadequate knowledge about exercise.

Regarding the extent of knowledge among patients regarding follow-up and periodic check-ups, 41 patients (54.7%) had inadequate information about periodic checkups. According the perspective of the investigator, RLS and ESRD patients often lack knowledge about follow-up and periodic check-ups due to fatigue, sleep disturbances, cognitive challenges, and limited focus on long-term care during medical visits. These factors reduce their understanding of the importance of regular monitoring. Notably, our results contrast with those of El-Kass et al. (21), who reported that 72.0% of the studied patients.

Considering the overall degree of knowledge that patients receiving hemodialysis had about RLS and ESRD. The study’s findings demonstrated that 47 patients (62.7%) of those receiving hemodialysis showed inadequate overall knowledge about RLS and renal disease. From the perspective of the investigator, this could be connected to various reasons such as age, living in the rural area and lack of previous experiences or information’s about renal disease and RLS undergoing hemodialysis, because the most of the patients under study had no family history. This outcome was consistent with the research conducted by Mahmoud et al. (38), which discovered that 72.0% of participants demonstrated insufficient information.

Concerning the RLS severity of the patients, 39 patients (52.0%) reported having severe sleep disturbance and 38 patients (50.7%) reported severe impact of RLS symptoms on mood disturbance. Additionally, about half of the research’s participants stated slight movement helped alleviate their RLS. This may be attributed to the frequent hemodialysis sessions, which contribute to iron fluctuations and a higher prevalence of anemia, known to exacerbate RLS severity, sleep disturbance, and mood impairment, thereby reducing symptom relief by movement. This outcome was in line with a study done by Mohammed Syam et al. (39), which reported about two thirds of the participants had severe RLS-related sleep disturbance, nearly two-fifths of the patients reported minor alleviation from RLS from moving around, which reported that 66.0% of the participants had severe RLS symptoms -related sleep disturbance.

As regards patients’ total severity level of RLS, the study’s findings demonstrated that 21 patients (28.0%) had moderate RLS, 8 patients (10.7%) had mild RLS, 32 patients (42.7%) had severe RLS, and 14 patients (18.6%) had very severe RLS. From the perspective of the investigator, the variation in RLS severity among the studied patients may be due to differences in anemia, iron levels, dialysis adequacy, and comorbidities. These factors influence dopaminergic dysfunction and uremic toxin buildup, leading to severe symptoms in some and milder forms in others. This outcome was consistent with the research conducted by Yaseen et al. (40), which discovered that 18.0% of the participants had minimal RLS, 38.0% had intermediate RLS, and 44.0% had severe RLS.

Concerning the whole Physical dimension of patients’ QoL, 39 patients (52.0%) exhibited poor physical dimension for QoL. This may be attributed to the combined physical burden of ESRD, RLS and hemodialysis treatment, which leads to fatigue, muscle wasting and anemia. This outcome was consistent with Tayea et al. (41), who discovered that 48.0% of the patients under study reported poor physical QoL.

Concerning the whole psychological dimension of patients’ QoL, 41 patients (54.7%) had poor psychological dimension for QoL. This may be attributed to the psychological dimension for QoL is negatively impacted by factors such as uncertainty about prognosis, fear of complications, financial burdens, and social role changes. This outcome was consistent with El-Kass et al. (21), which discovered that a poor mental health dimension in 52.0% of the respondents.

Regarding patients’ total level of social dimension for QoL, 37 patients (49.3%) had poor social dimension for QoL. This may be attributed to hemodialysis typically requires patients to spend around three to four hours per session, three times per week, plus additional travel and preparation time. This intensive regimen often interferes with social engagements, work, and family involvement. This finding was consistent with (21, 42), who discovered that hemodialysis patients’ perceptions of QoL were poorer in the social domains.

Regarding patients’ total level of spiritual dimension for QoL, 33 patients (44.0%) demonstrated a moderate spiritual dimension. This result may be due to the variations in religious practices, cultural norms, social support, and access to spiritual care services may contribute to differences in patients’ spiritual QoL. This result was consistent with (21, 43), which discovered that patients on hemodialysis had a moderate degree of religious and spiritual wellbeing. However, this result was inconsistent with the study conducted by Tayea et al. (41), which discovered that 90.0% of the participants exhibited high spiritual QoL.

Regarding overall QoL of patients, 36 patients (48.0%) demonstrated poor QoL. This outcome could be the consequence of social, psychological, as well as physical disability. The results indicated that these three dimensions were markedly affected, Although the spiritual dimension was found to be at an average level, this alone was insufficient to compensate for the significant deficits in the other dimensions. This result was consistent with Bagasha et al. (19), which discovered that 72.0% of the ESRD patients under study had lower QoL.

Findings from the present study demonstrated that the overall RLS severity and QoL among patients had a negative association with high statistical significance (r = −0.415, p < 0.001). From the investigator’s perspective, this outcome could be clarified by the fact that patients’ physical functioning, psychological well-being, and social activities are all directly impacted by worsening RLS symptoms like exhaustion, unpleasant leg sensations, and sleep disturbance, which ultimately results in a marked reduction in overall QoL. This outcome was in line with research by Fauzi et al. (22), which revealed a strong, statistically significant negative correlation linking patients’ QoL with overall RLS severity.

Regarding the finding of this study, a meaningful negative correlation was observed between overall RLS severity and overall degree of knowledge among the patients under study (r = −0.296, p = 0.056), though this association did not reach statistical significance. From the perspective of the researcher, this association may reflect that increased knowledge is linked to better therapy adherence, greater awareness of risk factors, and adoption of positive habits (e.g., sleep hygiene, diet, exercise), all of which are associated with lower symptom severity. This result was consistent with the study by Yıldız et al. (36), which also reported an inverse relationship between RLS severity and knowledge about the disease.

Regarding the finding of this research, a meaningful positive correlation was observed between patients’ overall degree of knowledge and QoL (r = 0.528, p < 0.029). From the perspective of the researcher, this association may be explained by the possibility that patients with higher awareness tend to better comprehend the illness, adhere to therapy, and engage in self-care practices, which are in turn associated with improved symptom control, fewer complications, and enhanced physical, psychological, and social well-being. The present finding was in line with the study by Alikari et al. (44), which found that improved QoL was significantly associated with increased knowledge.

### 4.1 Strengths and limitations

The present study has several noteworthy strengths. First, the utilization of the Kidney Disease Quality of Life Short Form (KDQoL-SF™) instrument exemplifies the use of a well-validated, multi-dimensional quality of life assessment tool that represents best practice in the measurement of health-related quality of life in nephrology research. Second, the study adhered to the highest standards of research ethics, having obtained institutional review board approval and documented informed consent from all participants, thereby ensuring the protection of patient rights and confidentiality throughout the investigation. Third, the study is among the first to assess the association between RLS severity and quality of life in patients with ESRD receiving hemodialysis in the HD unit of Banha University Hospital, Qalyubia, Egypt, providing early evidence regarding the clinical relevance of RLS severity in this population. Fourth, the findings hold significant clinical relevance by demonstrating that enhanced patient knowledge may represent a modifiable factor that could contribute to improving RLS symptoms and enhancing quality of life a finding with direct applicability in clinical nephrology practice. However, several limitations must be acknowledged when interpreting the findings. First, the use of a descriptive correlational design precludes inferences about causality and does not allow assessment of temporal changes over time. Second, the study was conducted in a single hospital and included a relatively small sample (n = 75), which may limit the generalizability of the findings. Future studies should involve multiple hospitals across Qalyubia and other governorates with larger sample sizes to improve the robustness and applicability of the findings.

### 4.2 Future research directions

Building on the findings of the present study, several promising avenues for future research are identified. First, longitudinal prospective cohort studies with 12–24 months of follow-up are needed to examine temporal associations and changes in RLS severity, patient knowledge, and quality of life over time. Such designs would help clarify the directionality of these relationships and assess whether improvements in knowledge translate into sustained reductions in symptom severity and enhanced QoL. Second, multicenter investigations incorporating teaching hospitals across different governorates of Egypt, along with international validation in diverse population demographics, would substantially strengthen the external validity and generalizability of the findings. Third, randomized controlled trials testing structured educational programs focused on RLS recognition, symptom management, and QoL improvement strategies using RLS severity and QoL as primary outcome measures are warranted to establish causal relationships. Fourth, qualitative research employing semi-structured interviews, focus group discussions, and other qualitative techniques would provide deeper insight into patients’ experiential perspectives, identify barriers to learning and self-management, and elucidate the nuanced relationships between RLS, knowledge, and QoL in the hemodialysis context. Fifth, future studies should examine mechanistic pathways linking dialysis adequacy indices, anemia parameters, iron status, and pharmacotherapeutic regimens to RLS severity, which would advance understanding of the underlying pathophysiology. Finally, the development and periodic implementation of standardized health education programs in dialysis units, coupled with dissemination of educational materials through mass media, is recommended to enhance patient knowledge and ultimately improve quality of life among hemodialysis patients with RLS and ESRD.

## 5 Conclusion

The current study concluded that 62.7% of patients had insufficient overall knowledge regarding restless legs syndrome (RLS) and end-stage renal disease (ESRD). Regarding RLS severity, 42.7% of participants had severe RLS, 28.0% had moderate RLS, 18.6% had very severe RLS, and 10.7% had mild RLS, while none (0%) were symptom-free. Moreover, more than half of the participants reported low quality of life (QoL). Among the study participants, QoL was negatively and significantly associated with overall RLS severity. Furthermore, overall knowledge was negatively and significantly associated with overall RLS severity, and QoL was positively and significantly associated with the overall degree of knowledge.

## Data Availability

The datasets presented in this study can be found in online repositories. The names of the repository/repositories and accession number(s) can be found in the article/supplementary material.
